# Surgical treatment of fibrous dysplasia in the proximal femur

**DOI:** 10.3892/etm.2013.987

**Published:** 2013-03-04

**Authors:** ZHICHAO TONG, WENTAO ZHANG, NING JIAO, KUNZHENG WANG, BO CHEN, TUANMIN YANG

**Affiliations:** 1 Department of Osteopathy, Xi’an Red Cross Hospital, Xi’an, Shaanxi 710054;; 2 Department of Orthopedics, The Second Hospital of Xi’an Jiaotong University, Xi’an, Shaanxi 710004, P.R. China

**Keywords:** proximal femur, fibrous dysplasia, curettage and bone grafting, osteotomy, internal fixation

## Abstract

The aim of this study was to summarize oncological and functional results and to investigate surgical treatment methods and efficacies by conducting a retrospective study of patients with fibrous dysplasia (FD) in the proximal femur. A total of 15 patients with FD in the proximal femur were selected. Among them, 12 cases were monostotic and 3 cases were polyostotic. In addition, 2 cases were accompanied by shepherd’s crook deformity. All cases received internal fixation following focus curettage and impaction grafting. Among them, valgus osteotomy was conducted for 2 cases of shepherd’s crook deformity. All patients were followed up for 12–32 months. For 2 patients with shepherd’s crook deformity in the proximal femur, the collodiaphysial angle was recovered after an orthopedic procedure following osteotomy. In addition, no patient presented with postoperative recurrent lesions. At 3 months following surgery, local bone resorption was visible in the bone grafting area. Between 8 and 12 months after surgery, the bones in the bone grafting area had healed, pain had disappeared and gaits were nearly normal. An effective internal fixation following thorough focus curettage and allograft bone transplantation is an effective method of treating FD in the proximal femur. For patients with shepherd’s crook deformity, it is also necessary to perform valgus osteotomy to recover hip joint function.

## Introduction

Fibrous dysplasia (FD) is a benign bone lesion resulting from congenital dysplasia of bone. It is characterized by fibro-osseous tissue replacing normal bone tissue (1,2). The incidence rate of FD is difficult to estimate. However, it is not rare in the clinic. It is reported that this disease accounts for 5–7% of clinical benign tumors (1,3,4). According to its clinical characteristics, FD is divided into 4 types: monostotic type, polyostotic type, McCune-Albright syndrome and Mazabraud syndrome (1,5). FD may occur at any age, without gender tendency. In general, the mean age of patients with monostotic FD presenting with clinical symptoms is 15 years old, and the mean age of patients with polyostotic FD is 30 years old (1). Common invasive skeletal sites include the long bones, ribs, maxillofacial skeleton and pelvis. The proximal femur is the most commonly affected site and its main symptoms include pain, deformity and lameness which seriously influence the functioning of affected limbs (6). Between April 2007 and January 2009, the authors conducted surgical intervention in 15 patients with FD of the proximal femur to correct deformity, eliminate symptoms, recover function and prevent pathological fracture according to symptoms and lesion size (7) in order to relieve pain and recover activity. During follow-up, efficacy for all patients was satisfactory.

## Patients and methods

### General data

Among the 15 cases, there were 9 males and 6 females, and their ages ranged from 16 to 32 years old. Among them, 1 case was 16 years old and 14 cases were between 18 and 32 years old. The mean age was 25 years old. Twelve cases presented with unilateral lesions and 3 cases presented with bilateral lesions. In addition, 12 cases were monostotic, and 3 cases were polyostotic. Among the 15 cases, 2 cases of FD in the proximal femur were accompanied by shepherd’s crook deformity. The collodiaphysial angles were 80 and 100°, and the femur lengths were 5 and 3 cm shorter than the contra-lateral length, respectively. The disease courses ranged from 2 months to 16 years, and the mean was 2 years. All presented with hip pain, 10 cases were able to walk without the support of crutches, 3 cases required the support of crutches to walk, the gaits of 6 patients were normal, and there was no history of surgical treatment of pathological fractures. The patients had no endocrine disturbances and X-ray, CT and MRI examinations were conducted prior to surgery. Lesion ranges were all >50% of the femoral medullary cavity: 3 cases involved the partial head of the femur, 15 cases involved the trochanter and 3 cases involved polyostotic lesions in ipsilateral ilia and tibiae. The postoperative pathological results all presented FD and 4 cases also had bone cysts. This study was conducted in accordance with the Declaration of Helsinki and with approval from the Ethics Committee of Xi’an Red Cross Hospital (Xi’an, China). Written informed consent was obtained from all participants.

### Internal fixation following curettage and bone grafting

**Figure 1:**
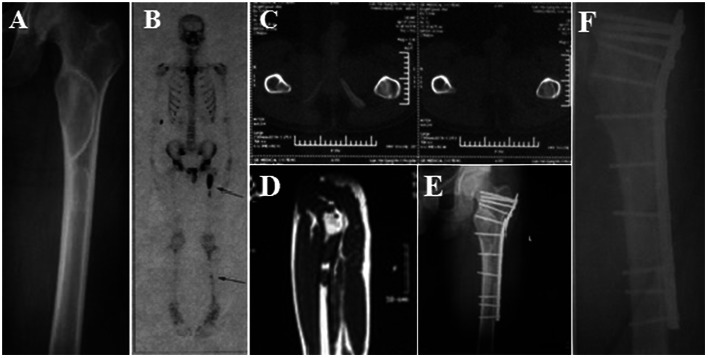
FD of the left proximal fumur of an 18-year-old man. (A) Anteroposterior preoperative radiograph; (B) preoperative radioisotope scanning; (C) pre-operative CT; (D) preoperative MRI; (E) anteroposterior postoperative radiograph; (F) anteroposterior radiograph shows bone union after 7 months of follow-up. FD, fibrous dysplasia.

The patients were placed in a supine position, the buttock of the affected side was cushioned and the proximal femur was exposed by approach from the hip and lateral upper femur. A window was opened at the anterolateral of the proximal femur FD lesions, and intramedullary gravel-like lesion tissues were removed with a curet under direct vision and were sent for pathological biopsy. The remaining tissues were washed, cauterized with an electrotome and soaked in 95% alcohol. A large block of autologous iliac bone (structural bone graft; Hubei Lianjie Company, Wuhan, China) was grafted at the femoral neck defect following curettage to improve the stability of internal fixation screws using iliac, and have better effects of repairing defects for autologous bone. (Fig. 1). For patients with an open osteoepiphyseal line, internal fixation of the anatomical plate was conducted in such a way as to avoid damaging the epiphyseal line.

### Osteotomy and internal fixation following curettage and bone grafting

**Figure 2:**
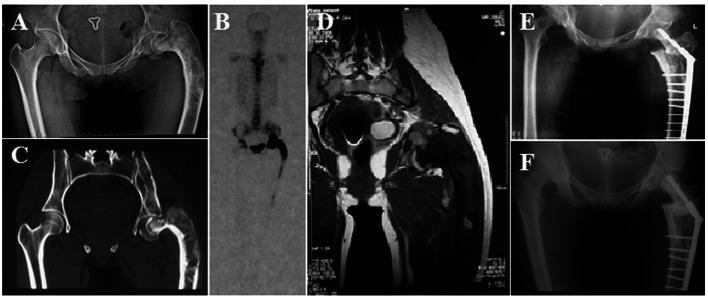
Polyostotic FD and shepherd’s crook deformity of a 28-year-old woman. (A) Anteroposterior preoperative radiograph; (B) preoperative radioisotope scanning; (C) preoperative CT; (D) preoperative MRI; (E) anteroposterior postoperative radiograph; (F) anteroposterior radiograph 4 years after sugery. FD, fibrous dysplasia.

For 2 patients with FD in the proximal femur accompanied by shepherd’s crook deformity, lesion tissues were removed with a curet under direct vision. After the remaining tissues had been cauterized with an electrotome and soaked in 95% alcohol, an allogeneic artificial bone was grafted by impaction. Valgus osteotomy was conducted in the lateral wedge nearby subtrochanteric femoral deformity vertex to relax gluteus medius and surrounding contractile tissues. After correcting the hip varus deformity or shepherd’s crook deformity and femoral axis rotational deformity, the 9-well dynamic hip screw (DHS) fixation was conducted (Fig. 2).

### Postoperative treatment

Long contraction exercises of the *quadriceps femoris* muscle and passive movement of the hip and knee joints began 24 h after surgery. Patients receiving the internal fixation after lesion curettage and impaction grafting were able to walk with the support of crutches at 8 weeks after surgery. For patients with FD in the proximal femur accompanied by shepherd’s crook deformity receiving osteotomy, internal fixation following curettage and bone grafting, the hip and knee joints were gradually unbent from the position of hip and knee flexion in order to avoid stretching the sciatic nerve following surgery. After 4 months, these patients were gradually able to walk with the support of crutches and they could walk without support of crutches after 10 months. In addition, postoperative follow-up and X-ray examination were conducted regularly.

## Results

### FD is confirmed by intraoperative histological examination of the excised specimens

All patients were followed up for 12–32 months, and postoperative incisions were healed in first grade healing. Following curettage and bone grafting, pain was relieved, 13 patients did not present postoperative recurrent lesions and no internal fixation became loose in the internal fixation group. In the bone grafting area, local bone resorption was visible at the 3rd month after surgery. From 8 to 12 months after surgery, the bones in the bone grafting area were healed, pain had disappeared, hip joint function was recovered and gaits were nearly normal. For the 2 patients with shepherd’s crook deformity in the proximal femur who received an orthopedic procedure following osteotomy, the collodiaphysial angle was recovered and femur length was increased by 3 or 4 cm. At the 4th month after surgery, patients were able to walk with the support of crutches, the pain had disappeared and hip joint function was recovered. In addition, all patients experienced no infection, re-fracture or progression of deformity, and no focus recurred during follow-up visits.

## Discussion

The development of FD lesions occurs at all stages of the bone formation and growth process, and is most likely to occur in the proximal femur. The treatment of foci at this site is more difficult. Body weight and mechanical stretching of muscle may cause stress and weaken bones, easily leading to fractures and deformity. In addition, repeated fractures may exacerbate the deformity. Among patients with multiple FD, a long bone usually fractures due to stress and thus bends to form a shepherd’s crook deformity (1). The age, focus size and behavior of the patient all influence the selection of a therapeutic regimen. For young patients, particularly patients younger than 12 years old, lesion symptoms prior to skeletal maturation are active, therefore the efficacy of surgical intervention is poor and the lesion is frequently recurrent or the nature of the lesion readily changes. We used oral bisphosphonates and followed up these patients (8). For polyostotic foci, lesions are likely to progress throughout adulthood, and the focus recurrence rate after curettage and bone grafting is higher. In this study, only 1 case was younger than 18 years old. According to the proximal femur symptoms and focus size, it is usually necessary to perform surgical intervention to relieve pain and recover activity. The purpose of surgery is to correct deformity, eliminate symptoms, recover function and prevent pathological fracture (7).

Lesion curettage and bone grafting is the main method of treating FD in the proximal femur, and highlights the lesion sites opening bone window method for the thorough removal of foci and applies electrosurgical cautery and 95% alcohol soaking for 20 min, which reduces the possibility of recurrence. Since we observed that bone resorption usually occurred at 3 months following focus curettage and allograft bone implantation, we used an impaction grafting technique to increase the bone density of the hollow bone graft. In general, there is a fracture risk following lesion curettage and the initial structural support role may be obtained by impaction grafting at the defect site following proximal femur lesion curettage. However, the bone graft will lose its structural strength as the bone bonding process progresses. The additional application of internal fixation may provide a strong mechanical support for the reconstructed defect bone to obtain early stability, which facilitates maintenance of gravity line of lower limb and the early ambulation of patients (without load or with partial load). There are numerous optional internal fixation methods (1,8–11). According to lesion range and surrounding bone quality, we used internal fixation with DHS/anatomical plates. For patients with an open osteoepiphyseal line, the internal fixation of the anatomical plate was conducted in such a way as to avoid damaging the epiphyseal line.

Among the 13 patients in the internal fixation group following curettage and bone grafting, pain was relieved, no case presented with postoperative recurrent lesions and no internal fixation became loose. At 3 months after surgery, local bone resorption was visible in the bone grafting area of 4 cases. At 8–12 months after surgery, the bones in the bone grafting area were healed, the myelocoele was unobstructed, pain disappeared, hip joint function was recovered and gaits were nearly normal.

Among the patients with multiple FD, a long bone usually fractures due to stress and thus bends into a shepherd’s crook deformity (2,12,13). The main symptoms of FD include pain of the affected hip, deformity, lameness and shortening of the affected limb. The purpose of the treatment is to obtain a normal gait and relieve the pain caused by secondary pathological fractures. However, orthopedic malformation is a challenge in surgical treatment (9–11,14). In this study, 2 cases of FD in the proximal femur were accompanied by shepherd’s crook deformity. The collodiaphysial angles were 80 and 100°, and femur lengths were 5 and 3 cm shorter than the contra-lateral length, respectively. The disease courses were 16 and 9 years, respectively. Both presented with hip deformity, pain and lameness. Under direct vision, an allogeneic artificial bone was grafted by impaction, and valgus osteotomy was conducted in the lateral wedge nearby subtrochanteric femoral deformity vertex to relax the gluteus medius and surrounding contractile tissues. After correcting the hip varus deformity or shepherd’s crook deformity and femoral axis rotational deformity, the 9-well DHS fixation was conducted to recover the collodiaphysial angle and femur length. Following surgery, the hip and knee joints were gradually unbent from the position of hip and knee flexion in order to avoid sciatic nerve stretch, the deformity was corrected and pain disappeared. It was observed that due to a larger bone graft mass, postoperative bone graft capacity was poor. At the 3rd month after surgery, there was a small amount of bone resorption at the grafting site. The patients were able to walk with the support of crutches 4 months after surgery and normally walk without the support of crutches after 10 months, the pain had disappeared and no deformity was observed. The purpose of the surgery was achieved and a good treatment efficacy was obtained.

For FD in the proximal femur, intralesional curettage and bone grafting is the main treatment mode (9,15). For bone defects following focus curettage, it is feasible to select autologous bone or allogeneic bone as the filling according to different situations. However, the issue remains a subject of debate. Stephenson *et al*(16) suggest that only the method of curettage and bone grafting is able to obtain a favorable result for patients older than 18 years old. However, Enneking and Gearen (17) reported that after focus curettage and autogenous cancellous bone transplantation were conducted for patients with FD in the proximal femur, the transplanted autogenous cancellous bone was completely replaced by hypogenetic bone tissue, tumors were likely to be recurrent and it was not possible to obtain a satisfactory efficacy, whereas, following the transplantion of autologous fibula into control cases, a satisfactory efficacy was obtained (18). The likely cause is that the cortical bone graft is rarely or slowly replaced by host bone and may be maintained longer. Therefore, it is more appropriate for FD treatment than an autologous bone graft. Certain scholars (19) suggest that the combination of focus curettage and cancellous bone or cortical bone grafting is not superior to simple osteotomy in FD treatment, while an autologous bone graft with blood vessels is not absorbed by the host bone (20). We suggest that as long as no further expansion of the tumor range or orthopedic failure occurs, it is acceptable for FD in the proximal femur following focus curettage, regardless of the bone graft material.

In conclusion, it is necessary to proficiently interpret the indications, conduct observations, administer oral bisphosphonates and perform curettage and bone grafting, internal fixation and orthopedic treatment of deformities in FD treatment. In addition, further clinical observations regarding the selection and application of bone graft materials are required.
